# Tackling Publication Bias in Clinical Trial Reporting

**DOI:** 10.1371/journal.pmed.0020367

**Published:** 2005-10-18

**Authors:** Emma Veitch, The PLoS Medicine Editors

## Abstract

PLoS announces a new journal devoted to publishing the results of clinical trials

Earlier this year, Richard Smith, former editor of the *BMJ* and a current member of the PLoS board of directors, provocatively suggested that “journals should perhaps stop publishing trials. Instead, the protocols and results should be made available on regulated Web sites” (DOI: 10.1371/journal.pmed.0020138). In the spirit of Smith's suggestion, we are launching *PLoS Clinical Trials* (http://www.plosclinicaltrials.org), a new “regulated Web site” (which we're still calling a journal) for peer-reviewed clinical trial reports.

We and the international advisory board believe this new journal is an important step toward overcoming publication bias, whereby published research differs systematically from unpublished data in its direction and strength of findings. Research funded by drug companies, for example, is less likely to be published than research funded by other sources (BMJ 326: 1167–1170), and roughly half of all completed trials are believed to go unpublished (AIDS Educ Prev 9 [Suppl 1]: 15–21). Clearly, bias in the published literature distorts the evidence available for other researchers, systematic reviewers, and ultimately for clinical decision making by health professionals and patients.

As we have previously argued (DOI: 10.1371/journal.pmed.0010046), many safeguards are needed to ensure a transparent trial reporting system, and *PLoS Clinical Trials* is just one such initiative. Universal prospective registration of trials in a publicly accessible repository is a crucial mechanism for ensuring that trials are known about at the start, to uniquely identify them and to reveal the existence of unpublished trials. Trial registration has gained widespread support from the major medical journals (JAMA 293: 2927–2929), and is currently the subject of a bill before the United States Congress (the Fair Access to Clinical Trials Act).

Two other initiatives include sponsors publicly releasing trial data on their own Web sites (BMJ 330: 479–480), and the US Food and Drug Administration posting selected review documents for new drug approvals on its Web site (DOI: 10.1371/journal.pmed.0010060). However, it is difficult to have confidence in data released by sponsors when the data have not been subjected to external, independent peer review. Furthermore, this information is not integrated with other data, or indexed.

*PLoS Clinical Trials* will be an unbiased venue for publication of trial data. The journal will peer review and publish the results of human clinical trials in all disciplines, and, crucially, direction of results, size, or significance will be no barrier to publication. Only an open-access journal could provide the business model for this type of publication. As an online, open-access journal that does not have to sell subscriptions or reprints to survive, *PLoS Clinical Trials* can consider all reports of trials that meet predetermined ethical and scientific criteria.

The rationale for open access to clinical trial data is overwhelming: many parties, from researchers to clinicians, meta-analysts to policymakers, have a need to read, analyze, and manipulate such data. Above all, patients who have altruistically volunteered to participate in trials want to be assured that the data are freely and publicly available for the benefit of others.

*PLoS Clinical Trials* will be online only. Each article will be cross-linked with trial registry records, such as those of ClinicalTrials.gov and the International Standard Randomised Controlled Trial Number (ISRCTN) Register. The inclusion of a trial identifier with every paper enables users to see clearly which results belong to which trial; without a straightforward way of identifying duplicate papers, treatment effects can be significantly overestimated. PLoS is collaborating with the Global Trial Bank (http://www.globaltrialbank.org), which is being developed by the American Medical Informatics Association, and which will ultimately ensure that trial data can be archived in a format that will allow scientists to analyze data across trials and, hence, generate new findings and insights (10.1371/journal.pmed.0020365).

We intend to go beyond CONSORT (a tool developed to improve the quality of reporting of randomized trials; http://www.consort-statement.org/), asking authors to not only submit a CONSORT checklist and flow diagram, but also to organize their papers according to the CONSORT structure. Readers will be able to quickly identify where in the paper they need to look to find out about a particular aspect of the design.

Rather than making recommendations about acceptance or rejection, peer reviewers of papers submitted to *PLoS Clinical Trials* will be asked to focus on improving the quality and transparency of trial reporting. Each trial report will be accompanied by an editorial summary of its strengths and weaknesses, including what it adds to current scientific knowledge. Readers will have the opportunity to post comments.

We offer unbiased reporting of results, but with rigorous external peer review, worldwide access free of barriers, and reports that are integrated with trial registries. We call on trial sponsors and investigators around the globe to support these goals. Pioneering authors should submit their work at http://www.plosclinicaltrials.org/.

